# Inferiority complex: why do sensory ion channels multimerize?

**DOI:** 10.1042/BST20211002

**Published:** 2022-02-15

**Authors:** Nikita Gamper, Shihab Shah

**Affiliations:** School of Biomedical Sciences, Faculty of Biological Sciences, University of Leeds, Leeds, U.K.

**Keywords:** G-protein-coupled receptors, nociception, pain, sensory neurons, TRPV1

## Abstract

Peripheral somatosensory nerves are equipped with versatile molecular sensors which respond to acute changes in the physical environment. Most of these sensors are ion channels that, when activated, depolarize the sensory nerve terminal causing it to generate action potentials, which is the first step in generation of most somatic sensations, including pain. The activation and inactivation of sensory ion channels is tightly regulated and modulated by a variety of mechanisms. Amongst such mechanisms is the regulation of sensory ion channel activity via direct molecular interactions with other proteins in multi-protein complexes at the plasma membrane of sensory nerve terminals. In this brief review, we will consider several examples of such complexes formed around a prototypic sensory receptor, transient receptor potential vanilloid type 1 (TRPV1). We will also discuss some inherent conceptual difficulties arising from the multitude of reported complexes.

## Introduction

Nociception is a complex physiological process in which damaging (or potentially damaging) conditions are detected by damage-sensing sensory neurons (nociceptors). Peripheral nerve endings of nociceptors transduce harmful environmental (or visceral) cues into action potentials and deliver these to the central nervous system (CNS) for processing. Higher CNS centeres translate this nociceptive input into the subjective feeling of pain. In most physiological scenarios, the first step in generation of a nociceptive signal occurs at the peripheral nerve endings of the nociceptive axons (or ‘fibers’) in close proximity to a damaging effector (e.g. fibers innervating fingertips of a person testing water in a pot). There are a multitude of sensory receptors (mostly ion channels) that open in response to certain physical changes in the immediate extracellular environment (e.g. sudden change in temperature or pressure) and depolarize the nerve terminal; different types of receptors sense different noxious signals. Upon activation, sensory ion channels generate ‘receptor potential’ and if strong enough, the receptor potential triggers action potential generation. There is an undeniable requirement for this process to be tightly regulated as a loss of control of pain signaling may lead to unwarranted pain which becomes a nuisance rather than an important asset for the body (e.g. as in chronic pain conditions). However, there are also scenarios where the activity of certain sensory receptors may need to be dialed up in order to increase the dynamic range of the sensory response, which, in turn, would allow the somatosensory system to better inform the body about the ever-changing physical environment. In many instances, control over the output signals generated by sensory receptors is achieved by additional protein partners forming various multi-protein complexes at the plasma membrane (PM) of sensory nerve terminals. In this brief review, we will consider several examples of such complexes formed around a prototypic sensory receptor, transient receptor potential vanilloid type 1 (TRPV1) and briefly discuss some implications and conceptual challenges arising from the multitude of reported complexes.

TRPV1 is a member of the transient receptor potential (TRP) channel family, containing 28 genes in mammals, most of these (including the *TRPV1* gene) encode Ca^2+^-permeable non-selective cationic channels [1]. Based on sequence similarity, TRP channels are subdivided into several families, including the Canonical (C), Vanilloid (V), Melastatin (M) and Ankyrin (A) types [1]. Many TRP channels are primary sensors responding to temperature changes (both heating and cooling), detecting various chemicals (including harmful ones), acidity, etc. [1,2]. Not surprisingly, several of these channel sensors are expressed in nociceptors. Perhaps the most well known and most studied of the TRP channels is TRPV1, the classical heat and chemical sensor of the body. TRPV1 is a versatile (or ‘polymodal’) sensor responding to such a diverse range of stimuli as noxious heat above 42°C, acidic conditions (low pH) and capsaicin, a component of chilli peppers (and this by no means is an exhaustive list of TRPV1 activation triggers). Recent advances in structural biology revealed these multiple modes of TRPV1 activation at atomic resolution [3,4]. Since TRPV1 is a non-selective cation channel, its activation commonly produces depolarization via influx of Ca^2+^ and Na^+^ and this depolarization, in turn, triggers nociceptive action potential firing. On top of this intrinsic versatility of channel activation mechanisms, TRPV1 is known to interact or multimerize with multiple other proteins, which further increases the sensory repertoire and dynamic range of sensory responses this channel generates.

## TRPV1–TRPA1 interactions

**Figure 1.**
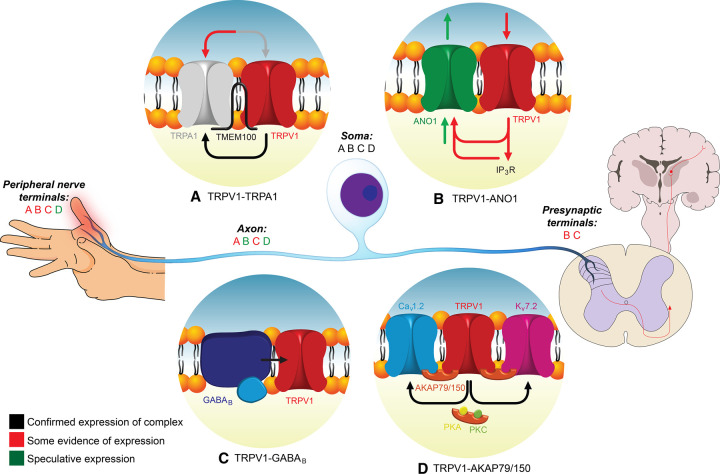
Overview of TRPV1-containing complexes and their localization along the peripheral somatosensory pathway. TRPV1–TRPA1 (**A**), TRPV1–ANO1 (**B**), TRPV1–GABA_B_ (**C**), and TRPV1–AKAP79/150 ‘supercomplex’ (**D**) are shown. Localization of complexes at various compartments (peripheral terminal, axon, soma, presynaptic terminal in the dorsal spinal cord) of the peripheral sensory neuron are shown with the corresponding letter for each complex, colour-coded to show level of evidence for expression at a specific compartment.

TRPA1 is another ubiquitous sensory channel and it was proposed to form multi-protein complexes with TRPV1 [5–7] (Figure 1A). The TRPA family of TRP channels was discovered in 1999 and is characterized by the presence of multiple (namesake) ankyrin repeats as part of the N-terminal domain [8]. TRPA1 is activated by a pungent compound, allyl isothiocyanate (AITC), produced by mustard plants, horseradish and wasabi [9]. In addition to AITC, TRPA1 can also be activated by a wide range of electrophilic compounds with irritant properties, including thiosulfinate, air pollutants, cigarette smoke and tear gas components, formaldehyde, reactive oxygen species (ROS) and many others [2,10-14]. In mammals, TRPA1 is expressed in small-diameter dorsal root ganglion (DRG) neurons and was found in 30% of TRPV1-positive neurons [15], as well as also being present in peptidergic nociceptors expressing calcitonin-gene related peptide (CGRP) and substance P-positive neurons [15]. Irritation, pain and itch produced by the compounds listed above are generally attributed to the activation of TRPA1 in nociceptors.

In addition to the range of irritants, TRPA1 was originally proposed to be activated by noxious cold (temperatures below 17°C); however, this property of TRPA1 is still controversial [16–18]. Interestingly, recent papers demonstrated that TRPA1, is actually one of the sensors responsible for sensing noxious heat together with TRPV1, TRPM3 [19] and, possibly, TRPM2 [20,21]. These findings, together with the observation that many TRPV1-positive neurons also express TRPA1, suggest some level of functional cooperativity and indeed, such cooperativity was long speculated [15]. Thus, TRPV1 activation by capsaicin was able to sensitize TRPA1 in trigeminal ganglion (TG) neurons and led to inflammatory cold hyperalgesia [22]. Conversely, TRPA1 activation was able to enhance TRPV1 activity via an increase in Ca^2+^ levels and in a protein kinase A (PKA)-dependent manner in sensory neurons [23]. A physical interaction between these proteins was also proposed; initially it was shown that TRPV1 and TRPA1 were able to form complexes in heterologous expression systems with studies showing that TRPV1 : TRPA1 concatomers behaved like TRPV1 channels, however, TRPA1 exerted an inhibitory effect on the activity of TRPV1 [24,25]. On the other hand, studies in TG neurons have suggested an inhibitory effect of TRPV1 on TRPA1 [5]. The physical interaction and formation of TRPV1–TRPA1 complexes was confirmed in sensory neurons and, interestingly, this assembly was shown to be controlled through the presence of an ‘adapter’ protein called TMEM100 [26]. This study demonstrated that when TMEM100 is not present, TRPV1 exerts an inhibitory effect on TRPA1 activity but the presence of TMEM100 enhances the activity of TRPA1 by spacing the channels [26]. Mutant TMEM100, containing mutations of three amino acids (KRR-QQQ) at the C-terminus, was unable to produce this spacing effect. Instead, this mutant enhanced the interaction within the TRPV1–TRPA1 complex. Furthermore, in TRPV1–TRPA1 expressing cells, mutant TMEM100 was able to reduce TRPA1 conductance [26]. This study concluded that TMEM100 allows control of TRPA1 interactions with TRPV1, thereby regulating the inhibitory influence of TRPV1 on TRPA1. TMEM100 had no effect on TRPV1 *per se*, presumably allowing it to continue to function physiologically, be modulated by TRPA1 through Ca^2+^ levels and be involved in other signaling cascades too. Of note, levels of TMEM100 also change in various pain-related conditions. Thus, complete Freund's adjuvant (CFA)-induced inflammation caused an increase in TMEM100 levels, whereas there is a reduction upon nerve injury [27]. These changes could be further investigated with regards to the effect on the TRPV1–TRPA1 complex and on the sensory outputs it generates.

Another recent study focused on how receptor sensitization of TRPV1 and TRPA1 was able to influence nociceptor sensitization and this was attributed to the TRPV1–TRPA1 complex itself [7]. Using reporter mice, it was found that neurons expressing both TRPA1 and TRPV1 were more susceptible to enhanced activity after various means of receptor sensitization, compared with neurons only expressing one of these channels, both *in vitro* and *in vivo* [7]. Furthermore, this study also investigated potential interaction sites on TRPV1 important for the formation of the TRPV1–TRPA1 complex; it was discovered that regions on the N-terminal domain and the TRP box containing region of the C-terminus of TRPV1 were responsible for the physical interaction between these channels [7]. Peptides encoding these regions were able to disrupt and reduce TRPV1–TRPA1 complex formation in sensory neurons [7]. The importance of this interaction towards the output signal generated by nociceptors was demonstrated by the ability of the peptides to reduce bradykinin-induced hyperalgesia [7]. Ultimately, it was concluded that these intimate interactions between TRPV1 and TRPA1 are important for sensitization of nociceptors.

The functional TRPV1–TRPA1 complex has recently been found in human odontoblasts [28]. TRPV1, TRPA1 and TMEM100 immunoreactivity was found colocalized in dental pulp tissue. Furthermore, there was also a functional interaction between TRPV1 and TRPA1 which was visualized using Ca^2+^ imaging experiments [28]. Further investigations are required to fully understand the activity of this complex in this tissue.

## TRPV1–ANO1 interactions

ANO1 (anoctamin 1, TMEM16A) is one of the latest additions to the sensory ion channel range. It is a Ca^2+^-activated Cl^-^ channel which has been implicated in inflammatory signaling in nociceptors [29–32] and, interestingly, was also suggested to be activated by noxious heat [33]. Most TRPV1-positive sensory neurons were found to express ANO1 [33,34] and like TRPV1, ANO1 was also implicated in the sensory response to bradykinin [29]. Application of this pro-inflammatory mediator was shown to activate ANO1 (via the inositol triphosphate (IP_3_)-induced Ca^2+^ release from the endoplasmic reticulum; ER) as part of the inflammatory response in DRG neurons [29,30,35]. Furthermore, the low Ca^2+^ sensitivity of ANO1 dictates local Ca^2+^ elevations are a requirement for activation of this channel under physiological conditions [29,36,37]. Interestingly, ANO1 displays a pattern of physiological triggers (bradykinin, heat) similar to TRPV1; additionally, it was shown to be functionally and physically coupled to TRPV1 in sensory neurons in a manner that amplifies TRPV1-mediated excitation and enhances pain [31,34]. A recent study [34] proposed that ANO1 and TRPV1 are physically coupled in DRG and Ca^2+^ entering through TRPV1 was able to directly activate ANO1 due to this proximity between channels, thereby overcoming the low Ca^2+^ sensitivity of ANO1 (Figure 1B). Accordingly, capsaicin-induced excitability of DRG neurons and *in vivo* nocifensive responses to capsaicin were reduced when ANO1 was blocked. This complex was also found centrally at the presynaptic terminals where ANO1 blockade was able to reduce glutamate release, therefore, a role in synaptic transmission has been touted for this complex (Figure 1B) [34].

However, the hypothesis of direct TRPV1–ANO1 coupling, whereby Ca^2+^ influx through TRPV1 activates ANO1, was recently refined. We suggested that this complex recruits IP_3_R-Ca^2+^ release to maximize ANO1 activation (Figure 1B). Of note, (i) TRPV1 activation induces phospholipase C (PLC) which cleaves phosphatidylinositol 4,5-bisphosphate (PIP_2_) and ultimately produces IP_3_R-Ca^2+^ release [31,38], and (ii) only ∼10% of the total TRPV1 current is Ca^2+^ current [39]. Hence, involvement of an additional, strong Ca^2+^ source would further increase the dynamic range of the TRPV1–ANO1 complex output (Figure 1B). Capsaicin application was indeed able to produce release of Ca^2+^ from the ER and depletion of the ER of Ca^2+^ resulted in a partial loss of ANO1 activation [31]. Close proximity between TRPV1, ANO1 and IP_3_R1 was also demonstrated using super-resolution microscopy and proximity ligation, suggesting the presence of multi-protein complexes consisting of all three proteins (Figure 1B). It must be noted that there was still a proportion of complexes consisting of ANO1 and TRPV1 only. It is tempting to speculate that TRPV1–ANO1–IP_3_R1 complexes may function to respond to inflammatory stimuli, whereas TRPV1–ANO1-only complexes would function as heat sensors, specifically. This would allow more efficiency, larger dynamic range and tighter control, all of which is very much required in pain-sensing neurons.

Another study reported that the menthol analog, 4-isopropylcyclohexanol, inhibited both ANO1 and TRPV1 and reduced membrane depolarization and action potential generation when applied to isolated DRG neurons treated with capsaicin [40,41], further strengthening the case for pharmacological targeting of the TRPV1–ANO1 tandem as potential therapeutic strategy.

## TRPV1–GABA_B_ complex

Recently, an unbiased proteomic method was used to identify interactions of TRPV1 with GABA_B_ receptors in sensory neurons, an interaction that reduces TRPV1 sensitization [42]. GABA_B_ receptors are inhibitory G-protein coupled receptors (GPCR) coupled to Gi/o α-subunits, which function to inhibit adenylate cyclase (AC), hence reducing cyclic adenosine monophosphate (cAMP) levels in the cell [43,44]. In its ‘classical’ form, this cascade also results in activation of inwardly rectifying K^+^ channels and inhibition of voltage gated Ca^2+^ (Ca_v_) channels in sensory neurons [44–46]. Using tagged-TRPV1 knock-in mice and pull-down proteomics, Hanack and colleagues [42] identified potential binding partners of TRPV1 in sensory neurons and found GABA_B1_ receptors amongst these proteins. A proportion of mouse DRG neurons were found to coexpress TRPV1 and GABA_B1_ receptors, which formed multi-protein complexes, as was further confirmed by immunoprecipitations and *in situ* proximity ligation. Intriguingly, activation of GABA_B_ receptors by its specific ligand, baclofen, did not affect TRPV1 activity *per se*, however, sensitization of TRPV1 produced by various types of inflammatory mediators, which work by activating different signaling cascades, was robustly inhibited by GABA_B_ activation [42].

Another point of interest is that the TRPV1–GABA_B_ complex lacked the GABA_B2_ subunit, which contains the G-protein signaling domain [44]. Additionally, GABA_B_-independent, Gs-GPCR-induced sensitization of TRPV1 was not affected by baclofen [43]. Both these observations indicate that modulation of TRPV1 by GABA_B_ was by a different means to what one would normally expect from inhibitory GPCR activation. Instead, it was proposed that GABA_B1_ receptors negatively modulate TRPV1 through direct interaction. It was further hypothesized that autocrine GABA release from nerve terminals is able to inhibit the sensitization of TRPV1 via GABA_B_ receptor activation (Figure 1C).

Recently, a functional interaction between TRPV1 and GABA_B_ receptors was reported in the presynaptic terminals in superficial layers of rat dorsal horn [47,48]. It was hypothesized that inhibition of presynaptic TRPV1 via presynaptic GABA_B_ receptor activation in the spinal cord mediates the anti-nociceptive effect of oxytocin.

## TRPV1–AKAP79/150 complexes

Another complexing partner of TRPV1 is the A-kinase anchoring protein (AKAP) 79/150 (human79/rodent150) [49]. AKAPs are scaffold proteins which were originally discovered as regulators of PKA and protein kinase C (PKC) [50] but were thereafter ascribed to scaffold and regulate a much broader list of targets [51]. PKA is activated through the cAMP signaling pathway, e.g. via Gs-GPCR activation [52], leading to AC stimulation which, in turn, produces cAMP and activates PKA. Phosphorylation of proteins by PKA initiates various effects on biological processes, with this being the critical aspect of some inflammatory signaling processes, including TRPV1 sensitization [53,54]. PKC on the other hand is a downstream effector of the Gq-GPCR pathway; it involves PIP_2_ cleavage by PLC and formation of IP_3_ and diaglycerol (DAG), with the latter able to activate PKC. Another major signaling pathway regulated by AKAP79/150 is CaN (Ca^2+^-calcineurin) and NFAT (nuclear factor of activated T-cells) mediated gene expression [55–59]. To tightly regulate these processes, AKAPs are involved with the formation of multi-protein signaling complexes and allow targeting of these various proteins to their targets within the neuron [60]. TRPV1 has been shown to be a binding partner for AKAP79/150, which orchestrates various further interactions and modulatory pathways targeting TRPV1 (Figure 1D) [53,54].

It has been extensively shown that TRPV1 activity is regulated by PKA, downstream of activation of different Gs-GPCRs such as prostaglandin E2 (PGE2) receptors, which leads to hyperalgesia [53,54,61,62]. Interestingly, inhibiting AKAP150 with a blocking peptide, Ht31, was shown to stop TRPV1 sensitization that normally occurs with PGE2 stimulation [63]. In mice, AKAP150 was expressed in over 80% of DRG also expressing TRPV1 and it was possible to coimmunoprecipitate both proteins as well [62], suggesting a physical interaction between the two. When the terminal 36 amino acids of AKAP150 were truncated, the interaction between TRPV1 and AKAP150 was lost, as was the PGE2-induced sensitization of TRPV1. The region in the TRPV1 C-terminus responsible for AKAP150 binding was also identified [54]. Importantly, PGE2-induced thermal hyperalgesia was severely reduced in mice with mutant AKAP150 (lacking the PKA-binding domain) knocked in (Δ36 mice) [62].

Recent work from Mark Shapiro's laboratory adopted a super-resolution microscopy approach to elucidate the ability of AKAP79/150 to act as a scaffold for multi-protein complexing in sensory nodose ganglia neurons [64]. Stochasitc optical reconstruction microscopy (STORM) can resolve proteins below the diffraction barrier with reported resolution <20 nm [65]. The study looked at the proximity (in a single neuron) between several ion channels, known to interact with AKAP79/150: K_v_7 (KCNQ) K^+^ channels, Ca_v_1.2 channels and TRPV1. Indeed, it was found that AKAP150 was able to cluster with either of the channels tested, suggesting the presence of multi-protein complexes consisting of AKAP150 with TRPV1, Ca_v_1.2 or, K_v_7.2, in various combinations, as well as ‘supercomplexes’ containing multiple channel types (Figure 1D). This clustering was lost in neurons from AKAP150 knock-out mice. But what could be the reason for this complexing? With regards to TRPV1, AKAP150 and K_v_7.2, the authors proposed functional coupling between TRPV1 and K_v_7.2 at membrane microdomains, rich in PIP_2_ (essential for K_v_7.2 activation). TRPV1-induced activation of PLC [31,38] would induce cleavage of PIP_2_, therefore, inhibiting anti-excitatory K_v_7.2 channel activity [64]. Such a mechanism would, again, increase the dynamic range of TRPV1-induced depolarization. With regards to TRPV1 and Ca_v_1.2 clustering, the tachyphylaxis of TRPV1 currents was found to be coupled to Ca_v_1.2 activity. When AKAP150 was lost from this complex, desensitization of TRPV1 was lost. One interesting aspect of AKAP150 signaling is its ability to homodimerize and allow the recruitment of a single PKA or PKC per AKAP150 molecule [66]. Thus, it would be interesting to see how such dimerization would impact on the arrangements within AKAP-mediated multi-protein complexes.

Interestingly, AKAP79/150 has also been shown to interact with another TRPV1-complexing partner mentioned earlier, TRPA1 [67]. Mechanisms of inflammatory mechanical hypersensitivity were found to involve activation of mGluR5 which sensitized TRPA1 in an AKAP-dependent manner [67]. Recent studies have shown that the C-terminus of TRPA1 acts as a potential signaling hub; binding of AKAP to TRPA1 potentiates channel activity at negative membrane potentials, either due to basal phosphorylation or via direct binding [68]. This opens up further lines of inquisition into TRPV1–TRPA1 interactions with regards to (i) sensitization by PKA and PKC, mediated by AKAP and (ii) the formation of this complex being orchestrated and scaffolded by AKAP. If the latter point is indeed true, this could further support the idea of AKAP as a ‘multitool’ scaffold for TRPV1-containing supercomplexes.

## Final remarks

Here we have considered several examples of regulation of the output signals generated by TRPV1 channels in sensory neurons by association with other channels and/or scaffolding proteins. A common theme in these associations is to increase or otherwise modify the dynamic range of the sensory response to a specific sensory stimulus. The list of examples discussed here is by no means exhaustive, thus direct interactions of TRPV1 with S100A1 [69], calmodulin [70], Src kinase [71], β-arrestin-2 [72], Sig-1 receptor [73] and SNARE proteins [74], have been reported.

**Figure 2.**
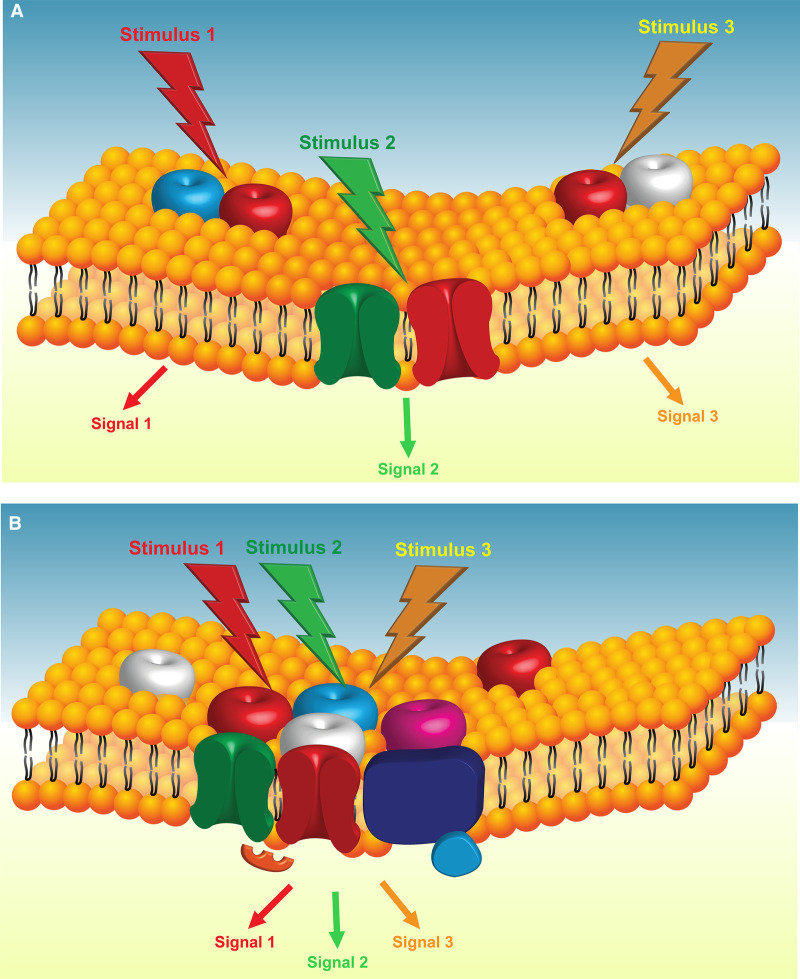
Association of a sensory ion channel into complexes with multiple different partners: two possible scenarios. (**A**) A principal sensory channel (red) forms individual complexes with other partner ion channels or regulatory proteins; each complex behaves independently. (**B**) A principal sensory channel forms a ‘supercomplex’ with multiple other partner channels/proteins.

This multitude of interactions reported in the literature raises a conundrum (Figure 2): do different proteins form individual clusters with TRPV1, where each complex is independent of other complex types (Figure 2A)? Or is there a larger dynamic system (or a ‘supercomplex’ alluded to above) where there is interplay between multiple, different proteins (e.g. TRPA1, ANO1, K_v_7, Ca_v_1.2 channels, etc.) with TRPV1 at the heart of it (Figure 2B)? If the first scenario is true — what governs the distribution of individual TRPV1 channels to different complexes? If the second scenario is true — how is it possible to generate specific signals to specific sensory stimulation? Inflammation, heat, capsaicin, etc. all result in TRPV1 activation and generate signals that often share some common features (e.g. heat-like sensation) but are not identical. Indeed, the first scenario (Figure 2A) would allow better matching of specific sensory stimulation to the particular type of output signal generated and would allow for specific complexes to be targeted to certain subcellular areas/structures. For example, TRPV1–ANO1 and TRPV1–TRPA1 complexes may be more relevant specifically at the peripheral terminals, while TRPV1–Ca_v_1.2 channel complexes may be better suited for more proximal compartments of a sensory neuron (soma, presynaptic terminals? Figure 1).

There is probably not a strict divide between these two scenarios (Figure 2A,B) and there may be a mixture of both principles of assembly of these complexes. For example, it could be that TRPV1 ‘supercomplexes’ assemble in a quasi-stochastic way, such that some of the following factors limit its composition: (*i*) not all of the proteins/channels discussed above would coexpress within a single neuron. (*ii*) Some of these proteins may have their own subcellular localization cues (i.e. K_v_7 channels are enriched at the nodes of Ranvier via ankyrin G binding [75]). (*iii*) Additional scaffolding proteins (which maybe specific to sensory neuron type or subcellular localization) may favour or exclude some of the partners. Furthermore, the composition of TRPV1-containing complexes could well be a dynamic process which switches from one state to another depending on the circumstances (e.g. due to AKAP79/150 dimerization or other triggers). For instance, there is some evidence that ER–PM junctions harbouring TRPV1–ANO1–IP_3_R1 complexes in DRG neurons [31] may indeed be dynamic as proximity between store-operated Ca^2+^ channel complex proteins (STIM1, Orai1) and an ER–PM junctional scaffolding protein, junctophilin-4, was induced by bradykinin [76].

Recent advances in cryo-EM have made it possible to relatively quickly obtain structures of an ion channel in multiple conformations and with different binding partners [3] and development of approaches such as CLEM (correlative light and electron microscopy) now makes it possible to identify individual proteins in their native cellular environment. Therefore, we believe that the near future will reveal answers at least to some of these exciting questions.

In conclusion, TRPV1 is a ‘prototypic’ sensory ion channel with intrinsically complex activation mechanisms. It can respond to a variety of sensory cues, most of which are related to adversity and may result in pain (including pathological). This already rich activation profile of TRPV1 is further enhanced by several levels of modulatory mechanisms, one of which is multimerization with other sensory ion channels, scaffolding and regulatory proteins. Understanding complex molecular interactions formed by sensory ion channels in nociceptive neurons may well allow new avenues of therapeutic intervention to be made available.

## Perspectives

- Molecular interactions of sensory ion channels, especially these involved with the detection of tissue damage, affect the way we experience pain. Understanding the complexity of these interactions may pave way for future pain treatments.
- Assembly of sensory ion channels into multimolecular complexes with other sensory and regulatory proteins may represent a way by which the dynamic range of sensory responses can be modulated to adapt to the changing environment.
- Thus far we have only scratched the surface of understanding the ‘sensory interactome’ but recent advances in structural biology (cryo-EM, AI-assisted protein folding predictions etc.) promise significant breakthroughs in the coming years.

## Abbreviations

AC: adenylate cyclase
AITC: allyl isothiocyanate
AKAP: A-kinase anchoring protein
cAMP: cyclic adenosine monophosphate
CNS: central nervous system
DRG: dorsal root ganglion
ER: endoplasmic reticulum
GPCR: G-protein coupled receptors
PGE2: prostaglandin E2
PIP_2_: phosphatidylinositol 4,5-bisphosphate
PKA: protein kinase A
PKC: protein kinase C
PLC: phospholipase C
PM: plasma membrane
TG: trigeminal ganglion
TRP: transient receptor potential
TRPV1: transient receptor potential vanilloid type 1
